# *Staphylococcus aureus* as an emerging model to study bacterial cell division

**DOI:** 10.1016/j.jbc.2025.110343

**Published:** 2025-06-06

**Authors:** Félix Ramos-León, Kumaran S. Ramamurthi

**Affiliations:** Laboratory of Molecular Biology, National Cancer Institute, National Institutes of Health, Bethesda, Maryland, USA

**Keywords:** DivIVA, PcdA, FacZ, FtsZ, orthogonal cell division, wall teichoic acid, lipoteichoic acid, sortase, Min system, Noc, McrB, AAA+, EVE domain, PBP3, RodA

## Abstract

Research on bacterial cell division has traditionally focused on rod-shaped model organisms, such as *Escherichia coli* and *Bacillus subtilis*. While these models have been important in uncovering broadly conserved factors involved in bacterial cell division, the assortment of bacterial shapes, cell wall structures, and lifestyles highlights the need to broaden the scope of study. This includes not only understanding how conserved mechanisms are adapted to diverse cellular morphologies but also discovering mechanisms that arise as specific adaptations to unique cellular shapes. In this context, alternative models such as *Staphylococcus aureus* are emerging to provide insight into how Gram-positive cocci overcome the challenge of lacking obvious cellular polarity to ensure accurate placement of the division septum and undergo binary fission. In this review, we highlight recent research that reveals how *S. aureus* performs several distinct but interrelated processes, including peptidoglycan assembly, placement of the cell division septum, and how the division septum can be used as a hub for modifying the peptidoglycan to decorate the cell surface of *S. aureus*.

*Staphylococcus aureus* is a monoderm spherical bacterium whose cell wall is composed of a thick layer of peptidoglycan (PG). *S. aureus* divides by binary fission, a process that, like in many other bacteria, relies on the tubulin homolog FtsZ (1, 2). In the presence of GTP, FtsZ polymerizes at mid-cell, forming a discontinuous ring-like structure termed the Z-ring (Fig. 1). FtsZ filaments exhibit dynamic treadmilling behavior, characterized by growth at one end through the addition of new subunits and shrinkage at the opposite end due to subunit release. This process is powered by GTP hydrolysis (3, 4). The arrival of FtsZ and other early cell division proteins at the division site initiates the recruitment of the machinery responsible for PG synthesis. This process ultimately leads to the formation of a septum that divides the cell into two identical daughter cells (Fig. 1) (5, 6). The decoration of PG with proteins that are cell wall-anchored, and other components such as wall teichoic acids (WTA), is tightly coordinated with PG synthesis at the division septum, thereby ensuring proper distribution of these components on the cell surface (7, 8, 9). In *S. aureus*, daughter cell separation occurs within milliseconds, due to the activity of specific PG hydrolases (10, 11). Interestingly, the next round of cell division highlights a curious spatial regulation of this process that was first described nearly 50 years ago: the daughter cells both undergo cell division in a plane that is roughly orthogonal to the division plane used by the parental cell (Fig. 1) (12, 13, 14). Although *S. aureus* cells were traditionally considered to remain spherical during the cell cycle, it is now known that *S. aureus* cells undergo a subtle elongation while cell division proceeds. This elongation is most obvious when septum synthesis is nearly complete, just before the separation of the daughter cells, resulting in cells that display short and long axes (and therefore a transient polarity; Fig. 1) as they approach cytokinesis (15, 16, 17). In this review, we will discuss recent advancements in understanding the molecular mechanisms involved in various stages of *S. aureus* cell division, including PG synthesis, the regulation of FtsZ positioning and dynamics, and the modification of the cell wall.

**Figure 1:**
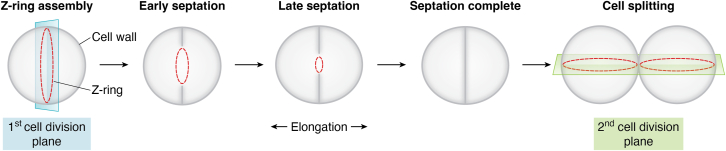
**Cell cycle of *S. aureus*.** Depicted is a spherical *S. aureus* cell that is dividing by binary fission (cell wall is *gray*). Cell division begins with the assembly of the Z-ring (*red*) at mid-cell, which defines the first division plane. The Z-ring facilitates the recruitment of the PG synthesis machinery that initiates septation. As the septum is synthesized inward, the Z-ring constricts. In addition to septal PG synthesis, PG is also synthesized at the periseptal regions, causing a subtle elongation of the cells, which becomes more apparent as the septum nears completion prior to cell separation (“Late septation”; center panel). Upon cell splitting, the Z-ring assembles along the next division plane, which is roughly orthogonal to the previous division plane.

## Cell wall remodeling in *S. aureus*

### Synthesis of peptidoglycan

**Figure 2:**
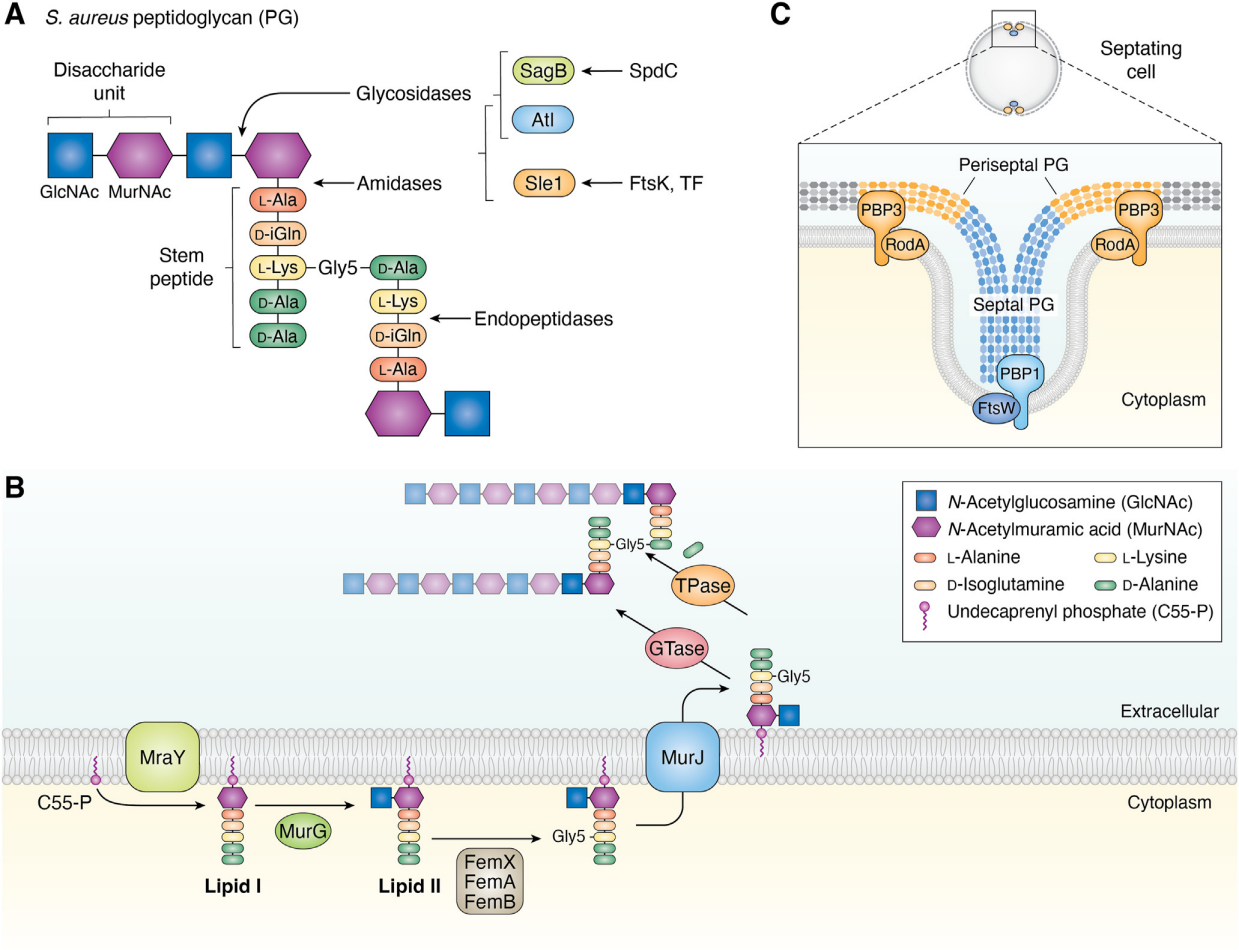
**Peptidoglycan composition and synthesis in *S. aureus*.***A*, *S. aureus* peptidoglycan is composed of repeating disaccharide units of N-acetylglucosamine (GlcNAc, *blue square*) and N-acetylmuramic acid (MurNAc, *purple hexagon*), depicted according to Symbol Nomenclature For Glycans (SNFG) standard (144). Each MurNAc is linked to a stem peptide consisting of L-alanine (*orange*), D-*iso-*glutamine (*light orange*), L-lysine (*yellow*), and two D-alanine residues (*green*). A pentaglycine (Gly_5_) bridge is attached to the L-lysine and cross-links to the fourth D-alanine residue through the activity of transpeptidases. The sites PG hydrolysis are also indicated. Proteins such as SagB and Atl exhibit glycosidase activity, cleaving the glycosidic bonds between GlcNAc and MurNAc within the glycan backbone. Amidase activity, exhibited by Sle1 and also by Atl, hydrolyzes the amide bond between the MurNAc and the stem peptide. Finally, other enzymes exhibit endopeptidase activity, cleaving the peptide bonds within the stem peptide or between cross-linked peptides. The activity of these hydrolases can be modulated by specific regulators. For example, SpdC regulates SagB, while FtsK and Trigger Factor (TF) contribute to correct secretion of Sle1 at the septum. *B*, peptidoglycan synthesis utilizes the lipid carrier undecaprenyl phosphate (C_55_-P, purple lipid). The process begins in the cytoplasm, where MraY catalyzes the addition of a MurNAc-pentapeptide to C_55_-P, forming lipid I. Next, GlcNAc is added to lipid I by MurG, producing lipid II. The Fem proteins (FemX, FemA, and FemB) sequentially add glycine residues to the L-lysine of the pentapeptide, generating lipid II-Gly_5_. This molecule is flipped to the outer leaflet of the membrane by the lipid II flippase MurJ. The glycosyltransferase (GTase) activity of PBPs transfers the disaccharide-stem peptide-Gly_5_ unit to existing glycan strands of PG. The transpeptidase activity (TPase) then catalyzes the cross-linking between the pentaglycine bridge and the fourth D-alanine residue of an adjacent stem peptide, releasing the fifth D-alanine. For simplicity, only a single cross-linking event between two glycan strands is depicted. *C*, two PG synthesis machineries operate during cell division in *S. aureus* leading to the synthesis of septal PG (*blue*) and periseptal PG (*orange*). The PBP1-FtsW complex (depicted in shades of *blue*) catalyzes the synthesis of septal PG (*blue* polymer) at the division plane building the septum that grows inwards. Concurrently, the PBP3-RodA complex (depicted in shades of *orange*) synthesizes PG at the periphery of the base of the septum, producing periseptal PG (*orange* polymer). This activity contributes to subtle elongation of the cells as the cell cycle progresses.

*S. aureus* is an excellent model for studying PG synthesis due to its relatively simple machinery and limited redundancy in components compared to other bacteria. PG consists of glycan strands cross-linked by short peptides. The glycan backbone is made up of repeating disaccharide units of N-acetylglucosamine (GlcNAc) and N-acetylmuramic acid (MurNAc). Attached to each MurNAc is a short peptide chain (stem peptide), which in *S. aureus* is composed of L-alanine, D-*iso*-glutamine, L-lysine, and two residues of D-alanine. In *S. aureus*, the glycan strands are cross-linked by pentaglycine (Gly_5_) bridges, which are attached to the L-lysine residue on one peptide chain and cross-linked to the fourth D-alanine residue of a neighboring peptide chain through the action of transpeptidases (Fig. 2*A*). The terminal D-alanine residue is typically released during the transpeptidation reaction while synthesis of new PG is happening (18, 19, 20, 21).

PG synthesis begins in the cytoplasm with the production of building blocks: GlcNAc and MurNAc-pentapeptide. The enzyme MraY catalyzes the attachment of MurNAc-pentapeptide to undecaprenyl phosphate (C_55_-P) in the inner leaflet of the cytoplasmic membrane, resulting in the formation of lipid I. Lipid I serves as the foundation for the next step, where MurG adds GlcNAc to the MurNAc residue, resulting in lipid II. The pentaglycine bridge is then added to lipid II through the action of the Fem proteins, which sequentially transfer glycine residues to the L-lysine residue using glycyl-tRNAs (22, 23). The modified lipid II is flipped to the outer leaflet of the membrane by the flippase MurJ (24), where lipid II acts as a substrate for penicillin-binding proteins (PBPs) to incorporate into the growing peptidoglycan layer (Fig. 2*B*) (19). PBPs perform two key activities: transglycosylation activity, which involves the incorporation of the disaccharide-pentapeptide unit from lipid II into the growing glycan chains, and transpeptidation activity, a reaction that forms cross-links between the peptide chains attached to the MurNAc residues. Class A PBPs are bifunctional enzymes that have both activities. In contrast, class B PBPs are monofunctional enzymes with only transpeptidase activity and require a cognate partner from the shape, elongation, division, and sporulation (SEDS) family, which possesses transglycosylase activity. A third group, class C PBPs or low-molecular-weight PBPs, function as carboxypeptidases (25). *S. aureus* possesses only one class A PBP, termed PBP2, and two class B PBPs: PBP1 and PBP3 (18, 25). PBP1 functions alongside its cognate SEDS protein, FtsW, while PBP3 operates with its cognate SEDS partner, RodA (15). Additionally, *S. aureus* has a class C PBP, PBP4, which exhibits D, D-carboxypeptidase activity as well as transpeptidase activity, playing a key role in the high degree of cross-linking of the *S. aureus* PG (26). In methicillin-resistant *S. aureus* (MRSA) strains, an additional class B PBP, PBP2a, is present, which displays a significantly lower affinity for β-lactam antibiotics (27, 28). In addition to the PBPs, *S. aureus* encodes two glycosyltransferases, SgtA and SgtB, that function without a cognate transpeptidase (*i.e.*, monofunctional glycosyltransferases) (29).

*S. aureus* was historically believed to synthesize PG exclusively at the septum during cell division, resulting in perfectly spherical cells that never elongate. However, more recent evidence indicates that PG synthesis also occurs at the cell periphery, which contributes to an increase in cell volume, and in the “periseptal region” (which lies at the base of the division septum where the division septum meets the cell periphery; depicted in orange in the septating cell in Fig. 2*C*) that drives the subtle cell elongation observed in the later stages of the cell cycle (5, 15, 30). The PBP1-FtsW pair is exclusively localized at the division septum and, along with PBP2, is responsible for synthesizing septal PG (15, 20, 31). The PBP3-RodA pair, in contrast, exhibits peripheral localization and is recruited to the division site after PBP1-FtsW and likely contributes to PG synthesis both at the cell periphery and the periseptal region. This periseptal localization of PBP3-RodA drives the slight cell elongation during septation. Notably, deletion of *pbp3* or *rodA* results in more spherical cells that do not elongate during the cell cycle (15). Thus, *S. aureus* is now understood to possess two distinct PG synthesis machineries: one dedicated to septal PG synthesis, coordinated by PBP1-FtsW and PBP2, and another involved in periseptal PG synthesis, resembling the elongasome observed in ovococci, that is coordinated by PBP3-RodA (Fig. 2*C*) (5, 32).

Studies using atomic force microscopy (AFM) have characterized the *S. aureus* cell wall structurally (33, 34, 35). The PG layer appeared as a porous mesh, with a lower density in the outer layers and increased density in the inner layers closer to the cytoplasmic membrane, where active PG synthesis takes place. At the septum, the PG layers formed concentric rings that became exposed after cell separation (33, 34); this pattern at the septum was likely due to the transpeptidase activity of PBP1 (31). AFM also revealed that the initial stages of septation involve the formation of thick peptidoglycan band, termed the “piecrust” (31, 34). It is hypothesized that the formation of the piecrust may result from PBP2 activity, as depletion of PBP1 disrupts septation but does not affect the development of the piecrust structure (31). Recent studies have demonstrated that the cell wall ultrastructure is similar between MSSA and MRSA strains under normal conditions. However, in the presence of β-lactam antibiotics, MRSA strains exhibit significant alterations in the septal peptidoglycan structure, transitioning from organized concentric rings to a dense disordered mesh with reduced pore size (35). This mode of septal PG synthesis enables MRSA strains to divide even when PBPs are inhibited. However, this is not solely due to PBP2A, since successful growth in the presence of high concentrations of β-lactams also requires additional mutations in so-called “potentiator” genes (*pot* mutations), such as *rpoB*, *rpoC*, or *relA* (36, 37). Thus, two pathways apparently contribute to high-level resistance to β-lactams in MRSA strains: PBP2A compensates for the loss of native PBP2 transpeptidase activity, while *pot* mutations compensate for the absence of PBP1 activity. The latter mechanism seems to be linked to the stringent response, although the precise molecular details remain unclear (35).

### Peptidoglycan degradation

Degradation of PG is as crucial as PG synthesis because it plays key roles in cell wall turnover, expansion, cell shape maintenance, and daughter cell separation. Enzymes involved in PG degradation and remodeling are usually referred to as PG hydrolases and can be classified based on their enzymatic activities. Glycosidases (with glucosaminidase activity in *S. aureus*) cleave glycosidic bonds in the glycan chain; amidases break the amide bond between MurNAc and the peptide stem; and endopeptidases cleave peptide bonds within the stem peptides and cross-links (38) (Fig. 2*A*). During growth, cell volume increase, and cell elongation, the cell wall must reduce its stiffness, which may be achieved by reducing PG cross-linking and/or reducing the length of the glycan chains (26, 39). In *S. aureus*, glycan chains are notably short, averaging only six disaccharides in length (40). While several glycosidases contribute to this, SagB appears to play the most significant role (39, 41). SagB activity is regulated by SpdC (also known as LyrA), an integral membrane protein that is a member of the CAAX protease family (42, 43). Although SagB harbors an intrinsic glycosidase activity *in vitro*, SpdC preferentially localizes at the septum *in vivo* and directly interacts with SagB to modulate SagB activity such that SagB produces glycan cleavage products of the correct length (42, 43). Thus, the SagB-SpdC complex functions as a PG-releasing factor, cleaving nascent PG from its membrane lipid anchor to enable its full integration into the cell wall. SdpC also interacts with WalK, a histidine kinase that is part of the WalKR two component system that regulates the expression of various PG hydrolases (42, 43, 44, 45).

PG degradation also plays a crucial role in daughter cell separation, which occurs remarkably quickly in *S. aureus* (10). PG hydrolases with amidase activity are important for this process. One of them is the major autolysin Atl, a bifunctional hydrolase with both glycosidase and amidase activities. Once secreted, Atl undergoes proteolytic processing to generate two functional fragments, each carrying a distinct enzymatic activity: amidase and glycosidase (46, 47). Atl localizes to the peripheral rim of the septal surface before cell separation, and this localization depends on teichoic acids (48, 49, 50). Atl glycosidase activity requires the prior removal of stem peptides by its amidase activity, resulting in a sequential process: the amidase fragment hydrolyzes cross-linked peptides, thereby exposing naked glycan strands that are subsequently processed by the glycosidase fragment (11). LytN, another bifunctional hydrolase, has also been implicated in cell separation. Its localization to the division septum is regulated by a YSIRK signal peptide (discussed below in “Decorating the cell wall during septation”) (51, 52). Another critical hydrolase involved in cell separation is the amidase Sle1, whose deletion significantly delays daughter cell separation (53, 54). Septal secretion and localization of Sle1 rely on the divisome protein FtsK and the chaperone Trigger Factor (TF). TF interacts with septal FtsK and unfolded Sle1 and facilitates Sle1 secretion through the septal Sec systems while protecting it from degradation by the ClpXP protease complex (55).

In sum, the coordinated activities of PG synthesis and hydrolysis promote the expansion and maturation of the cell wall and ultimately drive daughter cell separation. Precise timing and proper spatial organization of these dynamic processes ensure preservation of the characteristic spherical shape of *S. aureus* and maintenance of envelope homeostasis throughout the cell cycle. A central aspect of this coordination lies in the selection of the cell division plane and proper positioning of the cell division machinery, discussed in the next section, which ultimately dictates where new PG synthesis occurs.

## Division plane selection and positioning of the cell division machinery

Each generation of *S. aureus* cell divides along a plane that is roughly orthogonal to the division plane that was used by the parental cell in the previous round of division (12, 13, 14). Variations in this unusual pattern have been observed in other cocci as well (56, 57, 58). Among coccoid species, the cell biology of *S. aureus* has been extensively studied and serves as a model for understanding how spherical cells, which lack intrinsic polarity, can overcome the challenge of selecting the correct plane for cell division. Z-ring positioning is best studied in rod-shaped bacteria such as *E. coli* and *Bacillus subtilis*, in which placement of the Z-ring is regulated by two key systems, both of which are negative regulators: the Min system, which inhibits FtsZ polymerization (near the cell poles in *E. coli* and immediately adjacent to nascent division sites in *B. subtilis*), and the nucleoid occlusion system, which prevents Z-ring formation over the chromosome (59, 60). However, more recent studies have reported the increasingly widespread use of positive regulators of Z-ring placement in bacteria, such as in the ovococcal *Streptococcus pneumoniae*, the Gram-negative rod-shaped *Myxococcus xanthus*, and the filamentous actinobacterium *Streptomyces* (61, 62, 63). Thus, although negative regulation of Z-ring positioning was once considered the norm, bacteria are likely to adopt diverse mechanisms depending on their morphology and mode of growth.

**Figure 3:**
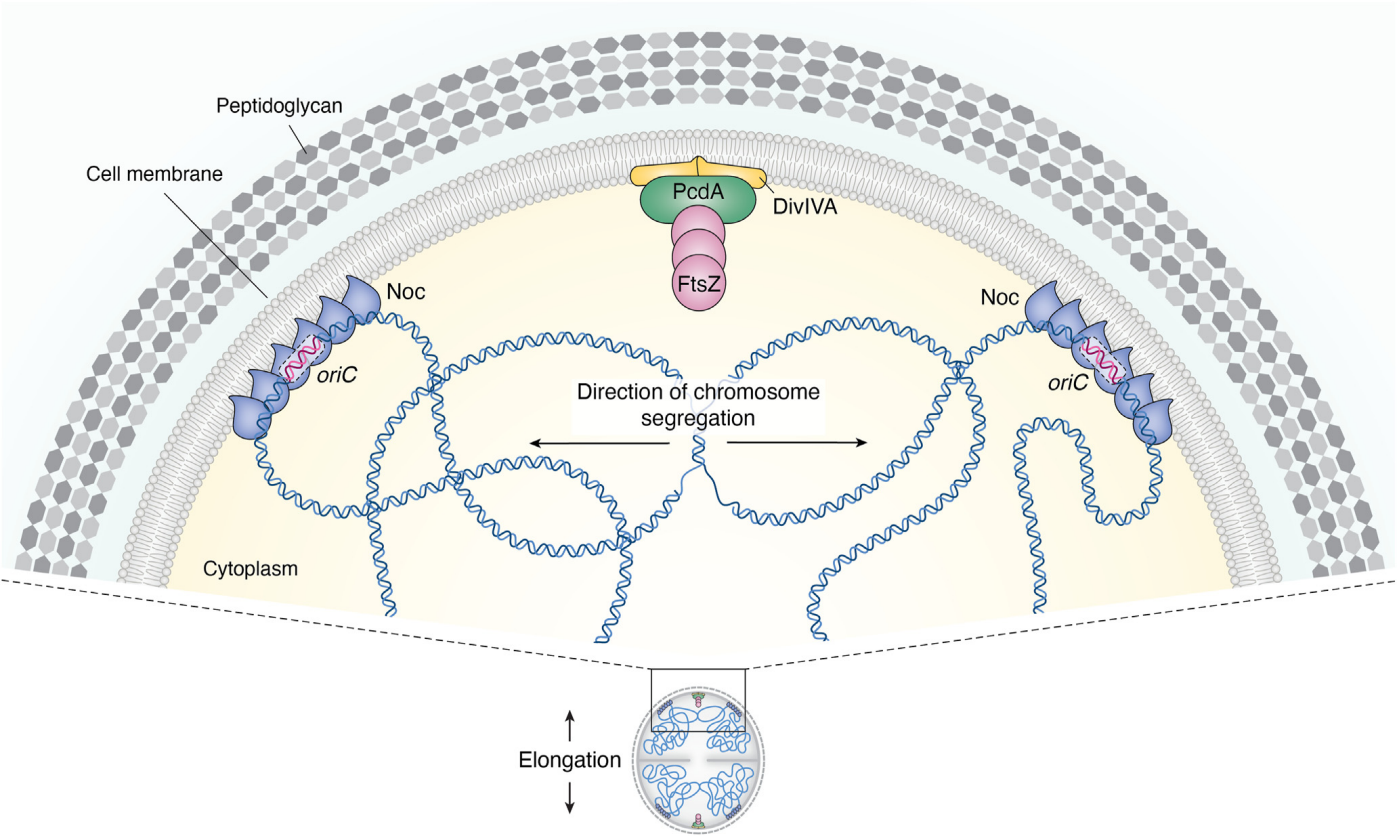
**Z-ring positioning in *S. aureus*.** The Noc protein (*blue*) binds to *oriC*-proximal regions of DNA and anchors the DNA to the membrane, preventing the assembly of cell division machinery in these areas. At the same time, the subtle elongation of the cell produces slightly increased micron-scale negative membrane curvature at the region orthogonal to the existing septum, where DivIVA (*yellow*) is recruited. PcdA (*green*) interacts with membrane-associated DivIVA and initially recruits unpolymerized FtsZ (*pink*) in an ATP-dependent manner; FtsZ then polymerizes along this axis, which is roughly orthogonal to that of the parental cell.

*Staphylococcus aureus* encodes a homolog of the nucleoid occlusion protein Noc, which plays a role in selecting the cell division site (14, 64, 65). In *B. subtilis*, Noc interacts with the chromosome near the origin of replication and binds CTP, triggering a conformational change that exposes an amphipathic helix, allowing it to interact with the membrane. It is hypothesized that subsequent CTP hydrolysis may facilitate the release of Noc from both the membrane and DNA (66). The tethering of chromosomal DNA to the membrane is essential for preventing divisome assembly over the chromosome (67). Although *S. aureus* Noc was assumed to function similarly to *B. subtilis* Noc, *S. aureus* Noc reportedly regulates the initiation of chromosome replication—a role that is not shared by its *Bacillus* counterpart and may participate in coordinating DNA replication and cell division (65). In *S. aureus*, *noc* is not an essential gene, and many cells in a *noc* deletion strain still undergo normal cell division, suggesting the existence of alternative mechanisms for Z-ring placement (64, 65). This additional mechanism is likely mediated by the PcdA protein, a positive regulator of Z-ring formation that is conserved in staphylococci and closely related genera (14). PcdA is an AAA+ NTPase of previously unknown function that is most closely related to the McrB family of GTPases (68, 69) and can hydrolyze ATP and GTP with similar turnover rates *in vitro* (albeit very inefficiently, due to extensive evolutionary degradation of its nucleotide binding pocket). In actively dividing cells, PcdA localizes to the mid-cell as a ring and co-constricts with the septum during cell division. Before septation is complete, though, a subpopulation of PcdA is redeployed to the new division site. PcdA interacts directly with both FtsZ and the scaffold protein DivIVA, the ortholog of which in *B. subtilis* reportedly preferentially embeds in membranes that display micrometer-scale negative curvature (70, 71). In the proposed model for Z-ring placement, DivIVA is first recruited to the next division site by a slightly increased local change in negative membrane curvature induced by cell elongation during the cell cycle. PcdA recognizes this future division site through its interaction with membrane-bound DivIVA and subsequently recruits unpolymerized FtsZ (likely in an ATP-dependent manner) to ensure assembly of the division machinery at the proper division plane (Fig. 3). A double deletion of *pcdA* and *noc* results in severe cell division defects, with most cells in the population unable to undergo orthogonal division. This suggests that the PcdA/DivIVA mechanism and Noc function in separate pathways to ensure accurate placement of the division site (14). Consistent with this model, PcdA localizes correctly to the next division plane even in the absence of the nucleoid, as observed in anucleate cells in a mutant impaired in chromosome segregation, indicating that PcdA localization is independent of the nucleoid (14). Furthermore, knockout of the cell elongation machinery (PBP3 and RodA), which results in *S. aureus* cells that remain largely spherical during the cell cycle, also results in an orthogonal cell division defect, with cells frequently failing to select the second division plane perpendicular to the previous one (14). Thus, instead of relying on a chemical landmark, such as a modification to the cell wall, to identify the next division plane, *S. aureus* instead exploits a transient cellular asymmetry that arises during normal growth to correctly localize the cell division machinery.

## Coordination of Z-ring constriction and PG synthesis machinery

The GTPase activity of FtsZ relies on its capacity to polymerize, as the nucleotide-binding pocket is localized at the interface where subunits interact. Thus, GTP hydrolysis is intrinsically linked to FtsZ polymerization, as contacts between monomers are essential for the catalytic process (72, 73). Staphylococcal FtsZ has been extensively studied using structural methods (74, 75). X-ray crystallography revealed that *S. aureus* FtsZ exists in two distinct structural conformations: open and closed. These conformations are thought to be linked to the polymerization state rather than the nucleotide-binding state. It is hypothesized that the closed conformation corresponds to monomeric FtsZ, while the open form plays a critical role in polymerization (75). Recent all-atom molecular dynamics simulations have revealed a greater complexity than previously assumed, demonstrating that FtsZ could transition between multiple states within polymers, including stable dimers where the subunits remain in a closed conformation (76).

FtsZ treadmilling plays a crucial role in cell division in the Gram-negative rod-shaped bacterium *E. coli* and facilitates the movement of inactive septal PG synthases, ensuring their proper distribution along the division site (77). However, this mechanism does not appear to apply to *S. aureus*. Previous research demonstrated that *S. aureus* cells treated with an FtsZ inhibitor, which blocks FtsZ dynamics, failed to form new septa. However, cells that had already initiated septation were still able to complete the process, suggesting that FtsZ dynamics are required only for the initiation of septation (20). Recent studies have shown that the septal PG synthases PBP1 and FtsW form a single population that moves more slowly than FtsZ treadmilling. This movement depends solely on PG synthesis and is independent of FtsZ dynamics (78). Interestingly, similar findings have been reported for PG synthesis machinery in the Gram-positive rod-shaped bacterium *B. subtilis* and the ovococcus *S. pneumoniae* (79, 80). These observations suggest that the machinery involved in septal PG synthesis in Gram-positive bacteria primarily exists in its active form, likely to support the synthesis of their thicker PG layer.

Because FtsZ lacks transmembrane domains, the Z-ring is anchored to the membrane by proteins that are also among the earliest to arrive at the division site. In *S. aureus*, the key membrane anchors for FtsZ are likely SepF and the conserved actin homolog FtsA, based on their conservation in other bacteria (81, 82, 83). These Z-ring anchors are important for the hierarchical recruitment of cell division proteins, and the regulation of FtsZ recruitment and dynamics could also play a crucial role during the early stages of cell division. In addition to its membrane anchoring activity, SepF promotes the stabilization of the Z-ring, possibly contributing to the alignment of FtsZ protofilaments (83, 84, 85). Recent studies have also demonstrated that the unfoldase ClpX is essential for promoting FtsA disassembly in *S. aureus*, and this regulatory mechanism is especially critical during growth at lower temperatures (86). Another key early cell division protein is ZapA, which is conserved across various bacterial groups. ZapA is not a membrane anchor, but it stabilizes FtsZ filaments by promoting bundling (87, 88). The specific role of *S. aureus* ZapA has not been studied; however, its conservation suggests it may function similarly to ZapA in *E. coli* and *B. subtilis*. EzrA is another early component that is conserved in Gram-positive bacteria and functions as a regulator of FtsZ (89). EzrA is required to prevent aberrant Z-ring formation and to promote the recruitment of the coiled-coil protein GpsB to mid-cell (90, 91). GpsB is a conserved protein in Bacillota that, in *S. aureus*, localizes to the division site and co-constrict along with the division septum (92). GpsB directly interacts with several components of the cell division machinery, including FtsZ, and enhances the GTPase activity of FtsZ (91, 92, 93, 94). The enhancement of FtsZ GTPase activity was initially puzzling since GpsB also displays FtsZ bundling activity (other FtsZ bundling proteins such as ZapA tend to inhibit the GTPase activity of FtsZ to stabilize FtsZ filaments (95)). However, *S. aureus* FtsZ, similar to *S. pneumoniae* FtsZ, displays a relatively high critical concentration for its GTPase activity *in vitro* compared to *E. coli* FtsZ (92, 96). Thus, a model that reconciles bundling and GTPase stimulatory activities is that *S. aureus* GpsB likely employs FtsZ bundling to increase the local FtsZ concentration to consequently stimulate the GTPase activity of FtsZ.

The crystal structure of *S. aureus* GpsB revealed that its N-terminal domain can homodimerize as a coiled-coil, like its ortholog in *B. subtilis* (94, 97). Interestingly, *S. aureus* GpsB exhibits unique features such as a 3-amino acid insertion that creates a hinge that may confer greater conformational flexibility compared to other orthologs. Mutations in this hinge impair GpsB function, indicating that flexibility is likely a key feature for GpsB to perform its function effectively (94). The N-terminal domain of GpsB interacts with the C-terminal region of FtsZ, which includes the sequence (S/T/N)RxxR(R/K). This sequence has been identified as a GpsB-binding motif in several GpsB interactors from other Gram-positive bacteria (94, 97, 98). Although GpsB was historically reported, primarily using the bacterial two-hybrid assay, not to interact with FtsZ in multiple organisms (99, 100, 101), recent work using purified proteins revealed that GpsB from *B. subtilis*, *Enterococcus faecalis*, *Listeria monocytogenes*, and *S. pneumoniae* interacts with its cognate FtsZ from that organism and stimulates FtsZ GTPase activity, although the requirement for the (S/T/N)RxxR(R/K) motif seems to be variable (98). In *S. aureus*, GpsB reportedly interacts with multiple other proteins, including the class C penicillin-binding protein PBP4, which is essential for PG cross-linking (94), TarG, a critical component of the WTA synthesis pathway (93), and PBP2, the primary class A penicillin-binding protein in *S. aureus* (17). Recently, a previously uncharacterized cell division protein termed FacZ was identified in a screen for *S. aureus* mutants that are impaired in envelope biogenesis. FacZ also interacts with GpsB *via* an (S/T/N)RxxR(R/K) motif (91). FacZ localizes to the plasma membrane and is enriched in the periseptal region but does not constrict with the septum as division progresses. Deletion of *facZ* resulted in pleiotropic defects in envelope biogenesis, including abnormal membrane and PG accumulations, as well as mislocalization of GpsB and FtsZ. However, the deletion of *gpsB* alleviated the defects caused by *facZ* deletion, indicating that FacZ both antagonizes GpsB activity and mediates the correct subcellular localization of GpsB (91).

The diverse protein partners of GpsB and their roles in different stages of cell division suggest that GpsB may act as a coordinator for FtsZ dynamics, cell wall synthesis, and WTA synthesis. Consistent with this model, recent studies have shown that deletion of *gpsB* results in cells that are rounder than wild type, indicating a defect in the subtle elongation that occurs during the *S. aureus* cell cycle (16, 17). In a methicillin-sensitive *S. aureus* (MSSA) strain (lacking PBP2A), deletion of *gpsB* led to increased septal localization of PBP3, the class B PBP directly involved in cell elongation (16). In contrast, in an MRSA strain, the absence of GpsB resulted in increased localization of PBP2 and PBP4 at the cell periphery. This suggests that the loss of GpsB disrupts the coordinated recruitment of these proteins to the septum, leading to increased peptidoglycan synthesis and cross-linking by PBP2 and PBP4 at the cell periphery. This peripheral synthesis may increase lateral cell wall stiffness, thereby impairing the elongation step and resulting in rounder cells (17).

The complex formed by DivIB, DivIC, and FtsL is part of the conserved cell division machinery that participates in recruiting the PG synthesis machinery in many bacteria (102, 103, 104). DivIB (“FtsQ” in *E. coli*) binds PG and is essential for *S. aureus* growth and septum completion (105). DivIC (“FtsB” in *E. coli*) is also essential for septal PG synthesis and interacts with several early and late components of the cell division machinery, including EzrA, PBP2, PBP1, and FtsW (90, 106, 107). Notably, DivIC is required for the spatial regulation of PG synthesis during septation, and its absence leads to defects in the recruitment of PBP2 and FtsW to the division site (106). The similarities in function between GpsB and the DivIB-DivIC-FtsL complex in coordinating cell growth and cell division begs the question of how these machineries may be linked, but to date, a functional relationship between them remains unknown.

Several other *Staphylococcus*-specific components have been identified as key players in coordinating cell division. One such component is SmdA, a septum-enriched membrane protein that is essential for growth in *S. aureus* (108). Depletion of the *smdA* gene resulted in cells with aberrant shapes and increased lysis. SmdA is an integral membrane protein characterized by an N-terminal transmembrane domain and a cytoplasmic domain containing a predicted nuclease-related domain (NERD). SmdA initially localizes at the cell periphery in pre-septating cells and accumulates at the septal region during septation. Interestingly, SmdA directly interacts with EzrA and the class B penicillin-binding proteins PBP1 and PBP3, suggesting a potential role in coordinating cell division and proper recruitment of the PG synthesis machinery. While the exact function of SmdA remains unclear, mutational studies indicate that its NERD domain is likely important for its activity (108). Additionally, two proteins, CozEa and CozEb (discussed in more detail below), have been also identified as interacting partners of EzrA (109, 110). However, CozEa and CozEb are not localized preferentially to the division septum; instead, they exhibit a highly dynamic spotty distribution throughout the membrane (110).

**Figure 4:**
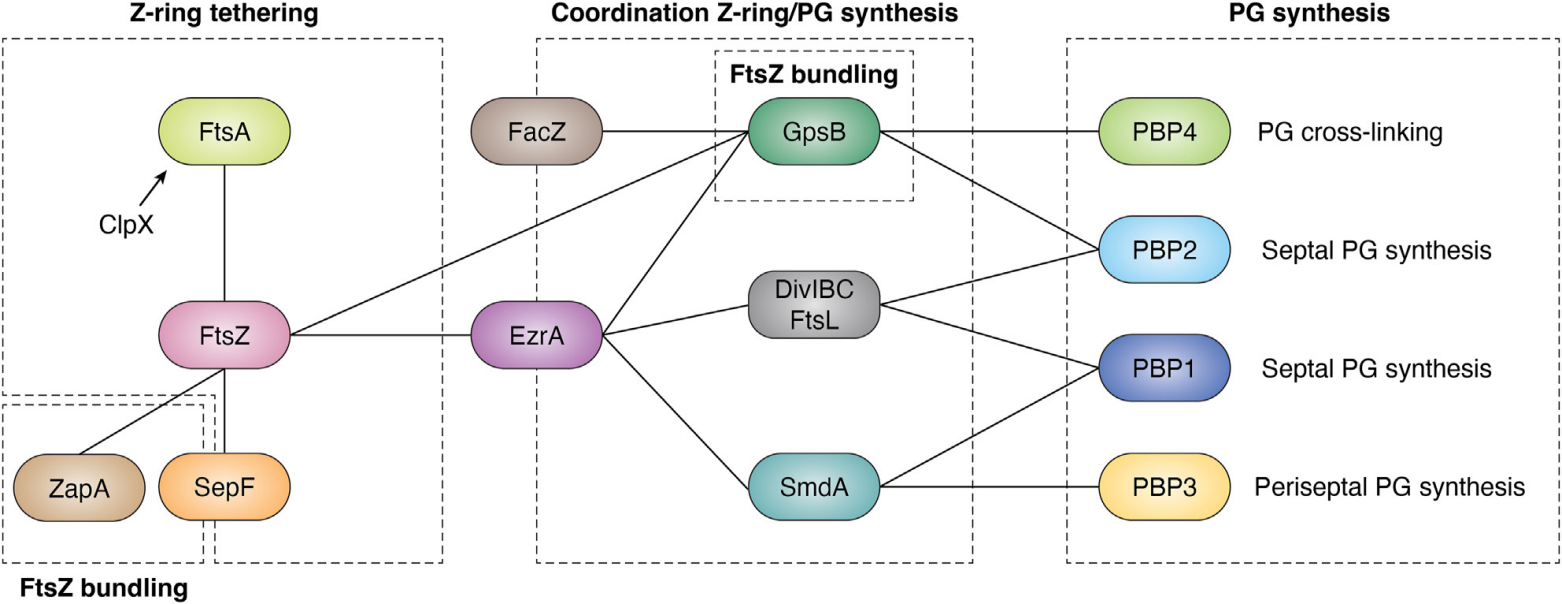
**Summary of interactions coordinating cell division and septation in *S. aureus*.** FtsA and SepF serve as a membrane anchors for FtsZ, and FtsA dynamics are regulated by the unfoldase ClpX. SepF and ZapA stabilize FtsZ polymers *via* FtsZ bundling activity. FtsZ directly interacts with EzrA and GpsB. GpsB regulates FtsZ dynamics *via* this interaction that resembles FtsZ bundling activity. EzrA facilitates the recruitment of key proteins through direct interaction, including GpsB, the DivIBC-FtsL complex, and SmdA. FacZ also interacts with GpsB to regulate GpsB activity. GpsB interacts with PBP4 and PBP2, potentially contributing to their recruitment to the division site. Similarly, the DivIBC-FtsL complex interacts with PBP2 and PBP1. SmdA interacts directly with PBP1 and PBP3, likely leading to the recruitment of the periseptal PG synthesis machinery.

Studies in *S. aureus* are therefore contributing to understanding how FtsZ dynamics and septal PG synthesis are coordinated in Gram-positive bacteria. FtsZ treadmilling appears to play a crucial role in the early stages of cell division, while the regulation of FtsZ-ring anchors, such as FtsA, by chaperones like ClpX, may help initiate the division process (78, 86). GpsB has emerged as a central regulator of FtsZ dynamics and the recruitment and distribution of PBPs at the division site that ensures maintenance of cell shape and drives the subtle cell elongation during the cell cycle that is required for proper selection of the cell division plane (16, 17, 92, 94). Additionally, newly identified divisome components such as FacZ and SmdA likely contribute to this process by regulating GpsB activity (91) or recruiting PBPs to the division site (108), respectively. A comprehensive summary of protein interactions discussed here that coordinate PG synthesis with FtsZ dynamics is shown in Figure 4.

## Decorating the cell wall during septation

### Teichoic acid synthesis

The PG component of the cell wall is decorated with various molecules, including teichoic acids (which may represent a larger fraction of the total mass of the cell wall compared to PG) and cell wall-anchored proteins, both of which contribute to regulating cell division, maintaining cell shape, protection against osmotic lysis, and facilitating interactions with the host (111, 112). Teichoic acids (TAs) are glycopolymers that can be attached either to the cytoplasmic membrane, where they are known as lipoteichoic acids (LTAs), or to the peptidoglycan, where they are known as wall teichoic acids (WTAs). TAs have zwitterionic properties due to the combination of negatively charged phosphate groups and D-Ala residues that may be esterified as a modification on the teichoic acid backbone, which introduces positively charged amino groups (111).

**Figure 5:**
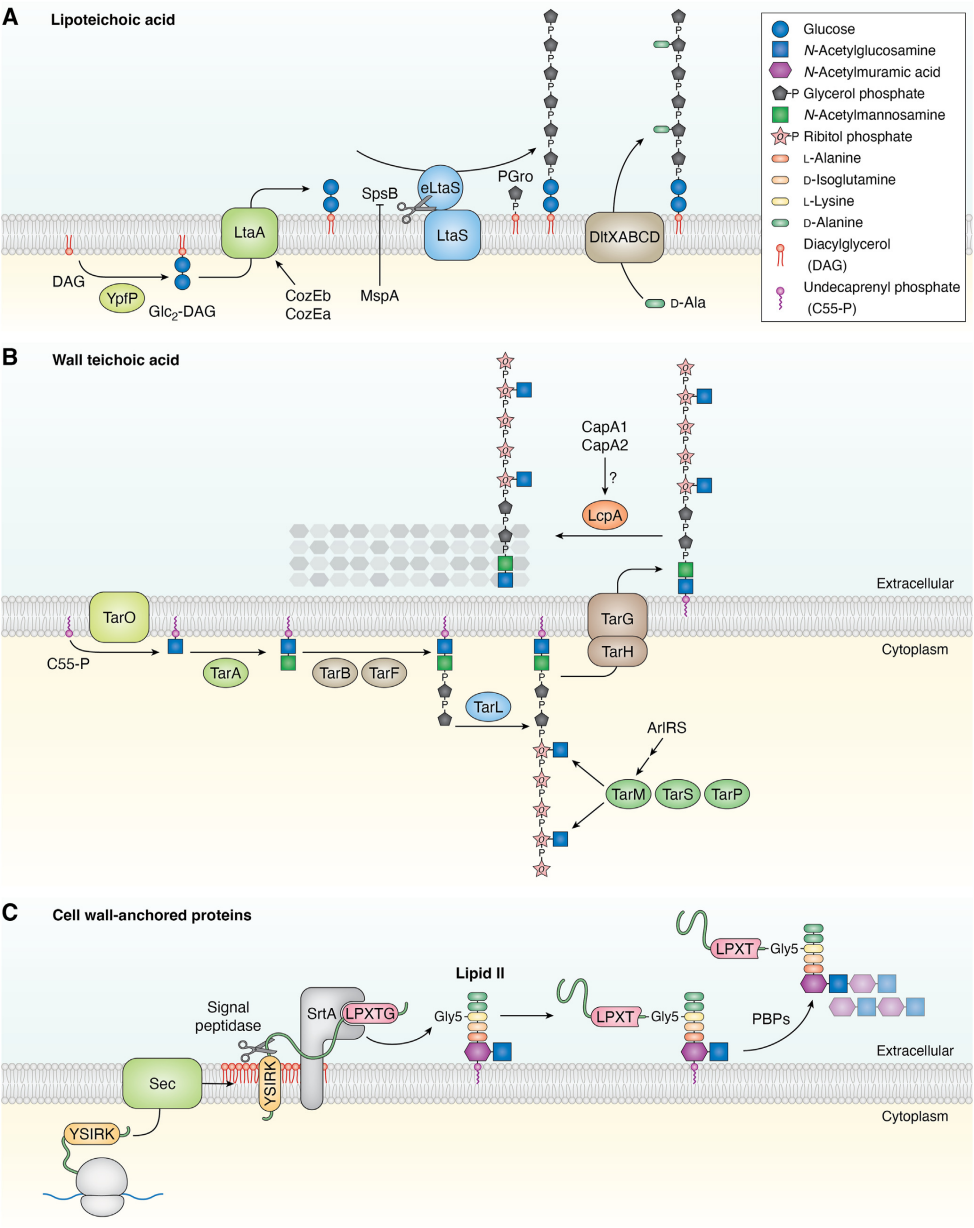
**Pathways for cell envelope modification in *S. aureus*.***A*, lipoteichoic acid (LTA) synthesis begins in the cytoplasm with the enzyme YpfP (*olive*), which adds two glucose units (*dark blue circles*) to diacylglycerol (DAG, *red* lipid), forming diglucosyldiacylglycerol (Glc_2_-DAG). LtaA (*light green*) flips Glc2-DAG to the outer leaflet of the membrane, with its activity regulated by the proteins CozEa and CozEb. LtaS polymerizes glycerol phosphate units (*blackoctagons*) onto Glc_2_-DAG using phosphatidylglycerol (PGro) as the glycerol phosphate donor (which produces diacylglycerol as a byproduct). Phosphate bonds are represented with a “P”. The activity of LtaS is controlled by its processing *via* the SpsB protease, which is inhibited by the protein MspA. Additionally, LTA is D-alanylated through the incorporation of D-alanine residues (*green capsule* shapes) by the Dlt system (tan; represented as a single complex). *B*, wall teichoic acid (WTA) biosynthesis uses undecaprenyl phosphate (C_55_-P, *purple* lipid) as a lipid carrier. TarO (*olive*) attaches a GlcNAc residue (*blue square*) to C_55_-P, followed by the addition of a ManNAc residue (*green square*) by TarA (*light green*). TarB and TarF (*tan*) subsequently add two glycerol phosphate units (*blackoctagon*) to the intermediate, and TarL (*blue*) polymerizes ribitol phosphate units (*light pink stars*). WTA can undergo further modification, including the addition of GlcNAc and other glycosylations, mediated by TarM, TarS, and TarP (*dark green*). The production of TarM is regulated by the AlrRS two-component signaling system. The WTA lipid precursor is transported to the extracellular space by the ABC transporter TarGH (*brown*). Lcp proteins, such as LcpA (*orange*), catalyze the incorporation of WTA into the PG. The specificity of Lcp proteins for WTA precursors may be regulated by CapA1 and CapA2. *C*, proteins containing a consensus YSIRK sequence motif (*gold* tag) within their signal peptide are directed to the Sec secretion system (*green*) at the septal membrane. It is hypothesized that DAG (*red* lipid), a byproduct of LtaS activity during LTA polymerization, may contribute to the recruitment of these proteins to the septal membrane. The signal peptide is cleaved by a signal peptidase, releasing the protein into the extracellular space. Proteins with a C-terminal sorting signal (LPXTG motif, *pink* tag) are recognized by the sortase enzyme SrtA (*gray*). StrA cleaves the sorting signal between the Thr and Gly residues and links the protein to the pentaglycine bridge of a lipid II molecule. PBPs facilitate the transfer of the protein bound to the PG precursor from the lipid carrier to the PG cell wall.

LTAs have a relatively simple composition and are anchored to the outer leaflet of the membrane *via* a diglucosyldiacylglycerol (Glc_2_-DAG) lipid anchor. The backbone of LTAs is composed of glycerol phosphate (Gro-P) units, with the 2-hydroxyl group of glycerol often substituted by D-Ala residues or N-acetylglucosamine (111). LTA biosynthesis begins in the cytoplasm, where the enzyme YpfP transfers two glucose residues from UDP-glucose to the DAG lipid, forming Glc_2_-DAG. This intermediate is flipped to the outer leaflet by the integral membrane protein LtaA (113, 114). Finally, the enzyme LtaS polymerizes Gro-P units, using the head group of the phosphatidylglycerol (PGro) lipids as substrate producing DAG as byproduct (Fig. 5*A*) (115). LTAs undergo further modification through D-Alanylation, a process mediated by the DltXABCD system (Fig. 5*A*) (116). Interestingly, the three enzymes involved in LTA synthesis interact with one another and with key cell division proteins, including EzrA, DivIB, DivIC, FtsL, FtsW, PBP2, PBP1, and PBP3 (117). However, while YpfP and LtaA are distributed throughout the membrane, LtaS is predominantly localized at the division septum, suggesting that while Glc_2_-DAG can be synthesized at various locations within the cells, LTA polymerization occurs preferentially during cell division (117). Mature LTAs are predominantly found at the peripheral membrane of dividing cells, with lower abundance at the septal region. This suggests a cell cycle-dependent LTA maturation process, wherein synthesis occurs at the septum *via* LtaS activity and LTA length gradually increases during cell division. Fully mature LTAs eventually cover the septal membrane just before the daughter cells separate (117, 118). Deleting *ypfP* and *ltaA* results in abnormally long LTA polymers, likely due to the use of alternative lipid anchors caused by absence and reduction of Glc_2_-DAC in the outer leaflet of the membrane, respectively (119, 120). The protein CozEb has recently been implicated in influencing the length of LTA polymers (110). Deleting *cozEb* results in the accumulation of long LTA polymers, similar to the effect observed with the deletion of *ypfP* and *ltaA*, but is not due to the mis-localization of the LTA synthesis machinery to the membrane (110). CozEb directly interacts with the flippase LtaA and modulates its activity, while the presence of its paralog, CozEa, counteracts the inhibitory effect of CozEb on LtaA activity. These findings suggest that CozEa and CozEb may regulate the distribution of Glc_2_-DAG in the outer leaflet of the membrane by modulating LtaA activity. However, their function likely extends beyond LTA biosynthesis, as the *cozEa cozEb* double mutant exhibits defects in membrane staining that are not observed in LTA biosynthesis mutants, suggesting a broader role for CozE proteins (110). Another level of regulation in LTA synthesis involves the processing of LtaS. LtaS contains five transmembrane segments and an extracellular C-terminal domain that harbors its enzymatic activity. The signal peptidase B (SpsB) processes LtaS by cleaving and releasing the catalytic extracellular domain (eLtaS) (121). The processing of LtaS has been shown to be coordinated with LTA synthesis (122). It is hypothesized that LtaS is processed by SpsB following its engagement with Glc_2_-DAG. The eLtaS fragment is less enzymatically active and requires the transmembrane domain for optimal LTA synthesis (121). This suggests that eLtaS likely remains associated with the integral membrane portion at the septal region until cell division is complete. Upon daughter cell separation, eLtaS is released, resulting in LtaS inactivation (122). Thus, regulation of SpsB may represent a critical step in controlling LTA synthesis. Recent work has further revealed that the membrane protein MspA interacts with SpsB and inhibits its activity by a hitherto unknown molecular mechanism, thereby impairing the LtaS processing (123).

WTA is another key component of the bacterial cell wall and is structurally more complex than LTA. WTA is anchored to the MurNAc residue of the PG *via* a disaccharide composed of GlcNAc and N-acetylmannosamine (ManNAc). This is followed by two Gro-P units and a variable number of ribitol phosphate (Rbo-P) units, although the exact composition varies across different *Staphylococcus* strains (124, 125, 126). For the synthesis of WTA, undecaprenyl phosphate (C_55_-P) is used as lipid carrier. The process begins with the integral membrane enzyme TarO, that transfers GlcNAc from UDP-GlcNAc to C_55_-P. Next, TarA adds a ManNAc unit using UDP-ManNAc as a donor, forming C_55_-PP-GlcNAc-ManNAc. Subsequently, the enzymes TarB and TarF add the first and second Gro-P units, respectively, using CDP-glycerol as a donor. The polymerization of poly-Rbo-P is then carried out by TarL, with CDP-ribitol serving as the donor (Fig. 5*B*) (126, 127). The enzymes TarM, TarS and TarP can further modify the poly-Rbo-P by glycosylation (128), which can vary depending on environmental conditions, as the expression of *tarM* is regulated by the two-component system ArlRS that depends on Mg^2+^ concentrations in the medium (129). The completed WTA polymers are then transported to the outer leaflet of the plasma membrane by the ABC transporter TarGH (124, 130). Once translocated, WTA is attached to the PG by enzymes of the LytR-CpsA-Psr (LCP) protein family (131, 132, 133). *S. aureus* encodes three LCP proteins (LcpA, LcpB, and LcpC) and deletion of all three genes is required to severely impair growth, suggesting some functional redundancy (132, 134). LcpC preferentially catalyzes the attachment of capsular polysaccharide (135, 136). Although the crystal structure of LcpA in complex with a lipid substrate revealed how these enzymes bind the substrate (133), it remains unclear how these enzymes specifically select the WTA intermediate over the PG precursor lipid II. Other proteins, such as CapA1 and CapA2, have been proposed to contribute to this selectivity, since CapA1 has been shown to stimulate LcpC activity and enhance specificity for WTA and capsular precursors over lipid II (136). In addition to mediating extracellular interactions at the cell surface with phages and host factors (7, 137), WTA influences various cellular aspects of *S. aureus* biology. For example, WTA has been implicated in controlling the activity and spatial distribution of PG hydrolases such as Atl which participates in cell separation and regulation of autolysis (49, 50, 138). Additionally, TarG interacts with the cell division protein GpsB, which raises the possibility that WTA may have a role in coordination of cell wall assembly and cell division (93).

### Cell wall-anchored proteins

*S. aureus* also decorates its cell wall by covalently anchoring proteins to the PG layer. These proteins are translocated across the cytoplasmic membrane and possess a LPXTG motif at their C-terminus, which is recognized by sortase A (SrtA) (139). SrtA cleaves the peptide bond between the Thr and Gly residues in the LPXTG motif and subsequently covalently links the cleaved protein to the pentaglycine bridge of a lipid II (140, 141), whereafter the protein will be incorporated into the growing PG by the action of the PBPs (Fig. 5*C*). SrtA substrates are proteins with diverse functions, including interactions with extracellular matrix proteins like fibronectin, fibrinogen, and collagen, as well as interactions with complement proteins and other components in host interactions (112). A subset of SrtA substrates harbor N-terminal signal sequences containing the YSIRK-GXXS motif (YSIRK signal), which directs their secretion to the division septum (Fig. 5*C*) and results in the abundant deposition and broad distribution of these cell surface proteins compared to the relatively low abundance of surface proteins secreted by canonical signal sequences and directed towards the polar peptidoglycan (9). The best-characterized YSIRK-containing surface protein is Staphylococcal Protein A (Spa) (118, 142). Secretion of Spa precursor at the septal region depends on the Sec system, which requires the activity of the ATPase SecA to power protein translocation; depletion of SecA blocks Spa targeting to the septal membranes (142). Several observations indicated a close connection between LTA synthesis and Spa secretion at the septal region (118, 122, 142). Mature LTAs localize to the peripheral membrane in dividing cells, whereas Spa secretion is restricted to the septal membrane (118). However, proper Spa trafficking at the septal membrane depends on the synthesis of Glc_2_-DAG by YpfP/LtaA and catalytically active LtaS (118, 122). It has been shown that the processing of LtaS regulates its activity, thereby controlling the timing of LTA polymerization during cell division. LTA polymerization involves the consumption of phosphatidylglycerol lipids, resulting in the production of DAG as a byproduct. This process would enrich septal membranes with DAG that could facilitate the recruitment of YSIRK precursors (122). Once proteins are secreted and anchored to the cell wall at the septum, they become exposed on the cell surface following daughter cell separation. The hydrolase Atl plays a critical role during cell splitting, and its activity is necessary for optimal surface display of Spa and fibronectin-binding proteins (FnBPs) that are also cell wall anchored. Consequently, disrupting autolysin function negatively affects *S. aureus* interactions with extracellular matrix components and leads to defects in the early stages of colonization in a mouse model (143).

The coordinated synthesis of teichoic acids and the spatially regulated insertion of surface proteins are therefore critical for maintaining cell envelope integrity and promoting host colonization during infection. These processes are tightly linked to the *S. aureus* cell cycle, ensuring that structural components and virulence factors are deposited at the correct place and time. Understanding how these pathways are integrated with the process of cell division (*i.e.*, division machinery placement and septal cell wall synthesis) provides a more comprehensive view of how *S. aureus* builds and remodels its cell wall during growth to ensure successful colonization.

## Concluding remarks

Studies on the cell biology of *Staphylococcus aureus* are revealing molecular mechanisms of cell division both that are unique to Gram-positive cluster-forming cocci, and those that are conserved across other Gram-positive bacteria. The simplicity of its cell division machinery, with minimal redundancy (for example, only four PBPs) provides an attractive model to study coordination between Z-ring constriction and PG synthesis. The discovery of two distinct cell wall synthesis machineries—one dedicated to septal PG synthesis and the other to peripheral PG synthesis—provides a relatively simple system to understand how two modes of growth can be coordinated in spherical bacteria. Additionally, *S. aureus* serves as a valuable model for studying the spatial coordination of cell division. Unlike rod-shaped bacteria, *S. aureus* lacks obvious natural polarity due to its spherical shape, offering unique insights into how coccoid bacteria achieve accurate division. Understanding the morphological changes throughout its cell cycle can also provide clues about cell cycle dynamics in other cocci that are less genetically tractable. Finally, research on *S. aureus* has historically advanced knowledge in areas such as cell wall modification, protein anchoring to the cell wall, and protein secretion, thereby providing a foundation for understanding these processes in other bacterial pathogens.

An important remaining challenge is to understand how the quizzical orthogonal cell division pattern of *S. aureus*, driven by transient cell elongation that leads to clustered growth of *S. aureus* colonies, contributes to the virulence of this organism. The complexity of cell division necessitates performing foundational experiments under laboratory growth conditions, but the consequences of alternating cell division planes for bacterial survival and colonization within a host are largely mysterious. Understanding growth advantages conferred by this mode of growth may reveal new strategies for disrupting key pathways that are essential for *S. aureus* pathogenesis. In this context, advances in imaging techniques to examine bacterial growth within mammalian tissues, as well as the development of *in vitro* models that better mimic host environments, will be instrumental in understanding the importance of modes of bacterial cell division in the context of specific infection niches.

## Conflict of interest

The authors declare that they have no conflicts of interest with the contents of this article.
